# Functional Connectivity Combined With a Machine Learning Algorithm Can Classify High-Risk First-Degree Relatives of Patients With Schizophrenia and Identify Correlates of Cognitive Impairments

**DOI:** 10.3389/fnins.2020.577568

**Published:** 2020-11-23

**Authors:** Wenming Liu, Xiao Zhang, Yuting Qiao, Yanhui Cai, Hong Yin, Minwen Zheng, Yuanqiang Zhu, Huaning Wang

**Affiliations:** ^1^Department of Psychiatry, Xijing Hospital, Fourth Military Medical University, Xi’an, China; ^2^Department of Radiology, Xijing Hospital, Fourth Military Medical University, Xi’an, China

**Keywords:** functional connectivity, machine learning, first-degree relatives, schizophrenia, cognitive impairments

## Abstract

Schizophrenia (SCZ) is an inherited disease, with the familial risk being among the most important factors when evaluating an individual’s risk for SCZ. However, robust imaging biomarkers for the disease that can be used for diagnosis and determination of the prognosis are lacking. Here, we explore the potential of functional connectivity (FC) for use as a biomarker for the early detection of high-risk first-degree relatives (FDRs). Thirty-eight first-episode SCZ patients, 38 healthy controls (HCs), and 33 FDRs were scanned using resting-state functional magnetic resonance imaging. The subjects’ brains were parcellated into 200 regions using the Craddock atlas, and the FC between each pair of regions was used as a classification feature. Multivariate pattern analysis using leave-one-out cross-validation achieved a correct classification rate of 88.15% [sensitivity 84.06%, specificity 92.18%, and area under the receiver operating characteristic curve (AUC) 0.93] for differentiating SCZ patients from HCs. FC located within the default mode, frontal-parietal, auditory, and sensorimotor networks contributed mostly to the accurate classification. The FC patterns of each FDR were input into each classification model as test data to obtain a corresponding prediction label (a total of 76 individual classification scores), and the averaged individual classification score was then used as a robust measure to characterize whether each FDR showed an SCZ-type or HC-type FC pattern. A significant negative correlation was found between the average classification scores of the FDRs and their semantic fluency scores. These findings suggest that FC combined with a machine learning algorithm could help to predict whether FDRs are likely to show an SCZ-specific or HC-specific FC pattern.

## Introduction

Schizophrenia (SCZ) is a devastating neurodevelopmental disorder with a complex genetic etiology (Lee et al., 2016). Multiplex family studies have established significant heritability for SCZ, which is often summarized as 81% (Light et al., 2014). First-degree relatives (FDRs; i.e., siblings, offspring, and parents) of patients with SCZ are 10 times more likely to suffer from SCZ than healthy controls (HCs) (Luykx et al., 2017). Consequently, when evaluating an individual’s risk for SCZ, the familial risk is among the most important factors (Pirjo et al., 2005). To define putative risk criteria for psychosis, prodromal criteria based on the Comprehensive Assessment of At Risk Mental States (CAARMS), the Criteria of Prodromal Syndromes (COPS), and the Bonn Scale for the Assessment of Basic Symptoms/Schizophrenia Proneness Instrument, Adult version (BSABS/SPI-A) have been validated in a range of studies (Yung et al., 2008; Schultze-Lutter, 2009; Woods et al., 2009). Early clinical intervention in SCZ has recently become a major objective of mental health services; those interventions include antipsychotic medications, cognitive behavioral therapy (CBT), and other novel strategies such as eicosapentaenoic acid (Addington and Heinssen, 2012). Because early intervention can help delay and prevent the onset of psychosis, the development of biomarkers allowing the early identification of individuals at elevated risk of developing SCZ is of great importance.

A previous large-scale network analysis showed that FDRs of patients with SCZ demonstrate similar deficits in connectivity metrics (Delawalla et al., 2008), interhemispheric functional connectivity (FC) abnormalities (Guo et al., 2014), default-mode network dysfunction (Meda et al., 2008), and rich club connectivity impairments, as do their relatives with SCZ (Zhang et al., 2016). This sharing of disease-specific patterns indicates that brain network disturbances are likely to show familial associations, possibly reflecting a vulnerability for SCZ. Consistent with these results, our previous study using stochastic dynamic causal modeling found similar anterior cingulate cortico-hippocampal dysconnectivity in unaffected FDRs and patients with SCZ (Xi et al., 2016). To date, however, the results of these studies showed minimal clinical impact for diagnostic and prognostic purposes, and traditional diagnostic and prognostic tools are still being used by psychiatrists. The most important reason is that the differences between FDRs and controls were reported at the group level, which provided limited information to make inferences at the level of the individual (Orrù et al., 2012).

With the continuous innovation of machine learning technology, pattern classification algorithms have become widely used in SCZ research. Previous studies showed that functional brain connectivity patterns can be used not only to classify patients with SCZ from normal controls but also to predict the development and prognosis of the disease. With the use of functional brain networks derived from an independent component analysis of resting-state functional magnetic resonance imaging (RS-fMRI), FC patterns reached an accuracy of 85.5% for distinguishing SCZ patients from HCs (Jing et al., 2019). Additionally, using a support vector machine (SVM) algorithm, the accuracy of FC patterns for differentiating controls from patients can reach 83.8% (Fan et al., 2011). More importantly, the classification scores obtained from the SVM could predict the prognosis, with high classification scores being associated with worse treatment effects.

In the present study, we investigated the classification efficiency of FC obtained from RS-fMRI for distinguishing SCZ patients from HCs, applied the classification models to determine whether FDRs were similar to SCZ patients or HCs, and finally explored whether the classification scores were able to predict the cognitive performance of the FDRs.

## Materials and Methods

### Subjects

The current study was approved by the First Affiliated Hospital (Xijing Hospital) of the Fourth Military Medical University. Written informed consent forms approved by the local Research Ethics Committee were signed by all participants. The study sample consisted of 40 first-episode SCZ patients from early intervention services within the outpatient clinic and inpatient department of Xijing Hospital, 36 FDRs of patients with SCZ, and 40 HCs recruited from the local community by advertisements. Two senior clinical psychiatrists diagnosed SCZ using the *Diagnostic and Statistical Manual of Mental Disorders* (Fourth Edition) (DSM-V) structured clinical interviews (SCIDs). All SCZ patients had a first episode with exposure to antipsychotic treatment within 2 weeks. Some patients with <6 months’ illness duration were diagnosed as FE-SCZ after a 6-month follow-up according to diagnostic criteria. The severity of symptoms was assessed using the Positive and Negative Syndrome Scale (PANSS) (Kay et al., 1987). Exclusion criteria consisted of (1) other DSM disease; (2) a history of treatment with transcranial magnetic stimulation, transcranial current stimulation, or behavioral therapy; (3) substance abuse; (4) other neurological diseases; and (5) pregnancy or other MRI contraindications. Additional exclusion criteria for the HCs included a current or past history of psychiatric illness and the presence of psychosis in FDRs (Yuan et al., 2018a).

### MRI Acquisition

All MRI data were collected on a 3.0-T Siemens Magnetom Trio Tim scanner at the Department of Radiology of Xijing Hospital. During data acquisition, participants were asked to keep their eyes closed, to let their mind wander, and to keep awake (Liu et al., 2013; Yuan et al., 2017a). A head coil fitted with foam pads was used to minimize head motion, and earplugs were used to dampen scanner noise (Liu et al., 2017; Yuan et al., 2017b). Resting-state functional scans were acquired with an echo-planar imaging (EPI) sequence using the following parameters: repetition time (TR), 2,000 ms; echo time (TE), 30 ms; field of view, 220 × 220 mm; matrix, 64 × 64; flip angle, 90°; number of slices, 33; slice thickness, 4 mm; 240 volumes; and a total of 7 min. After acquisition of the RS-fMRI, a high-resolution T1 image was acquired for anatomical reference using a magnetization-prepared rapid gradient-echo sequence with the following parameters: TR, 2,530 ms; TE, 3.5 ms; flip angle, 7°; field view, 256 × 256 mm; matrix, 256 × 256; slice thickness, 1 mm; slice gap, 0 mm; slices, 192; resolution, 1 × 1 × 1 mm; and a total of 6 min 30 s.

### Data Preprocessing

Preprocessing of the RS-fMRI data and the calculation of FC measures were performed in a similar manner to those described in previous studies (Zhu et al., 2019; Song et al., 2020). The first 10 images were discarded to ensure MRI data stability (Yuan et al., 2016), and then the remaining 230 images were slice timing corrected and realigned to the first image, during which the average frame-wise displacement (FD) was obtained (no differences in this were found across the three groups; see Table 1). Inter-scan motion was assessed using the translation and rotation parameters, and an exclusion criterion of >2.5 mm translation and/or >2.5° rotation in each direction at each time point was set. Two SCZ patients, two HCs, and three FDRs met the criteria and were excluded from further analyses, resulting in 38 SCZ patients, 33 FDRs, and 38 HCs for final inclusion. As FC measures are sensitive to head motion, Friston-24 parameters were used to regress out their effects. To further reduce the effects of nuisance factors, signals from cerebrospinal fluid and white matter were also regressed out. The global signal was not removed as suggested in a previous study (Hahamy et al., 2014). Then, the DARTEL toolbox was used to normalize the data into Montreal Neurological Institute (MNI) space (Ashburner, 2007), and the resulting images were finally smoothed with a 6-mm full width at half maximum (FWHM) Gaussian kernel.

**TABLE 1:** Demographic and clinical features of the participants.

Demographic and clinical features of the participants	SCZ	FDR	HC	
		
	*n* = 38	*n* = 33	*n* = 38	
		
Characteristic	Mean (± SD)	Mean (± SD)	Mean (± SD)	*p*
Age (years)	26.3 ± 6.9	26.7 ± 9.6	25.4 ± 5.6	0.32
Gender (male/female)	19/19	18/15	19/19	0.84
Education level (years)	13.5 (2.96)	12.1 (4.15)	14.2 (3.37)	0.12
Age at onset (years)	19.2 (3.77)			
Length of illness (years)	2.72 (2.95)			
Frame-wise displacement	0.26 (0.11)	0.23 (0.11)	0.20 (0.09)	0.13
PANSS score				
Total	22.6 ± 5.8			
Positive	21.5 ± 8.1			
Negative	44.1 ± 6.2			
General	85.8 ± 12.8			
Semantic fluency scores	12.1 ± 6.2	14.9 ± 5.6	16.7 ± 4.3	0.001

*Differences in age, education levels, frame-wise displacement and semantic fluency scores were carried with one-way ANOVA. Differences in gender was carried with Chi-square test.*

### Functional Connectivity of the Whole Brain

The Craddock atlas was used to parcellate the whole brain into 200 regions of interest (ROIs) (Craddock et al., 2012). This new atlas has been validated that it can successfully parcellate group resting-state fMRI data into spatially coherent functionally homogeneous clusters of the network (Allen et al., 2014). The time series within each region were first band-pass filtered (0.01–0.08 Hz) and then averaged. For each participant, FC was calculated between each ROI using Pearson’s correlation coefficients, resulting in 19,900 [(200 × 199)/2] dimensional FC feature vectors for each subject.

### Feature Selection

Before the classifier model was built, an initial feature selection step was performed for data dimension reduction. The current study used an F-score for feature ranking, which was shown to be an effective method in previous studies (Liu et al., 2015). Leave-one-out cross-validation (LOOCV) was used to evaluate the performance of the classifier. In LOOCV, one subject is used as test the data, and the classifier is trained on the remaining dataset. For each LOOCV iteration, the features were ranked from the highest to lowest according to their F-score, and the first 644 features (see details in subsection “Overall Classifier Performance”) were used to build the classifier.

However, for each iteration of the LOOCV, the data subset used for feature ranking was a little different, and the final features selected for the classification model differed slightly between each iteration. Therefore, consensus features were identified, with these being the features that were always selected to build the classification model in each iteration of the LOOCV. The weight for each consensus feature was defined as the average of the weights across the 76 LOOCV iterations. A weight for each ROI was also calculated by summing one half of the consensus feature weights associated with that region, which represented the ability of that region to discriminate SCZ patients from HCs.

### Classification and Support Vector Machine

The SVM algorithm was selected for classification because it has shown good efficiency when the sample size studied is relatively small. Patients with SCZ were labeled as 1, and HCs were labeled as −1, and a decision function was determined during the training step and used to predict the labels of the test data. To avoid overfitting and to allow direct extraction of the feature weights, a linear kernel SVM was implemented using the LIBSVM toolbox (Chang and Lin, 2011), with the parameter C set to the default value of 1. With the use of the LOOCV strategy, the accuracy, sensitivity, specificity, and area under the receiver operating characteristic (ROC) curve (AUC) were obtained, and the statistical significance of the accuracy was assessed using a permutation test (Golland and Fischl, 2003).

### Classification of First-Degree Relatives

The above-mentioned classification model was used to classify SCZ patients and HCs, and our next analysis was to investigate whether the final classification model could be used to determine whether the FDRs showed similar FC patterns to SCZ patients or HCs. After the classification model was built, the FC of each FDR was input as test data into each iteration of the LOOCV, to obtain its corresponding prediction label (1 or −1). Therefore, each FDR was given 76 individual prediction labels. The classification score, which is the average of the 76 prediction labels, was used as a robust measure to characterize the similarity of each FDR’s FC pattern to an SCZ pattern (in the range of −1 to 1, a positive score indicated an SCZ pattern).

### Correlation Analysis Between Cognitive Function and Classification Scores

Finally, a general linear model was used to investigate the correlations between classification scores and measures of cognitive function in FDRs, with age, sex, and years of education as covariates. A semantic fluency test (animal version) was administered to evaluate the executive function and the semantic memory, which are severely affected in SCZ; the performance was analyzed using the number of correct words within 1 min.

## Results

### Demographic and Clinical Data

The demographic and clinical data are shown in Table 1. No significant difference was present between the SCZ patients, FDRs, and HCs in any of the demographic variables, including age, sex distribution, education level, and FD. However, significant differences were found for semantic fluency scores across the three groups.

### Overall Classifier Performance

**FIGURE 1:**
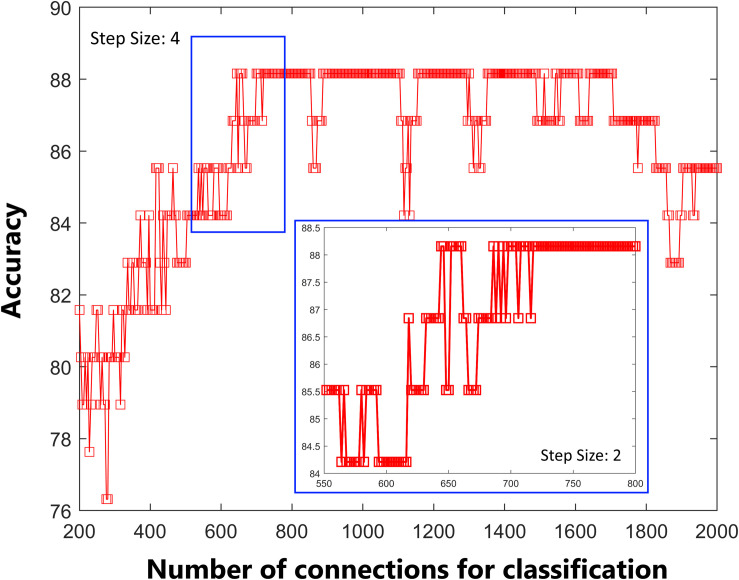
Predictive accuracy as a function of the number of connections used in the classification process. The connections were ranked according to F-scores in descending order.

**FIGURE 2:**
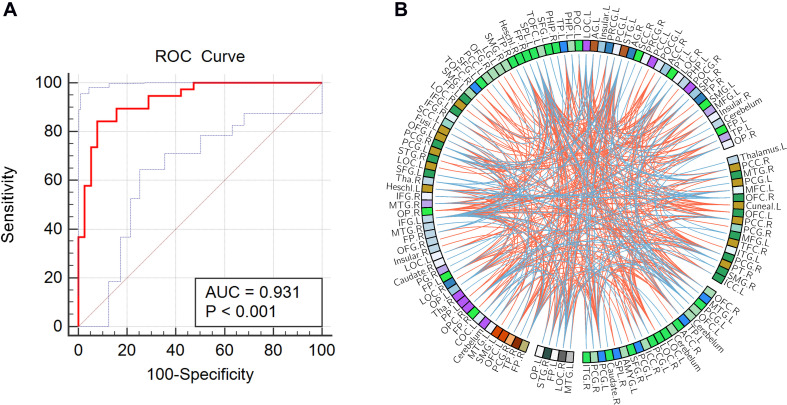
**(A)** Receiver operating characteristic (ROC) curve of the classifier; the gray line indicates the 95% confidence interval for the area under the ROC curve. **(B)** The consensus functional connections. The brain regions are represented by a square on the circumference of the big circle. The lines connecting two squares represent the connections between the corresponding two brain regions. The *red lines* represent positive connections, and the *blue lines* represent negative connections.

As shown in Figure 1, the accuracy of the linear SVM classifier reached up to 88.15% (84.06% for sensitivity, 92.18% for specificity, and *p* < 0.001 by permutation test) using the 644 highest-ranked FC features. Thus, we selected the top-ranked 644 features in each iteration of the LOOCV for the classification features. The discriminative score for each tested individual was acquired from the SVM classifier and an ROC curve was created (Figure 2A), which showed an AUC of 0.93, indicating good classification power. A non-linear SVM classifier was also trained and showed similar results; however, to reduce the risk of overfitting and to directly calculate and exhibit the FC weights and ROI weights, the following analysis is based on the linear SVM classifier.

### Consensus Features and Region Weights

**FIGURE 3:**
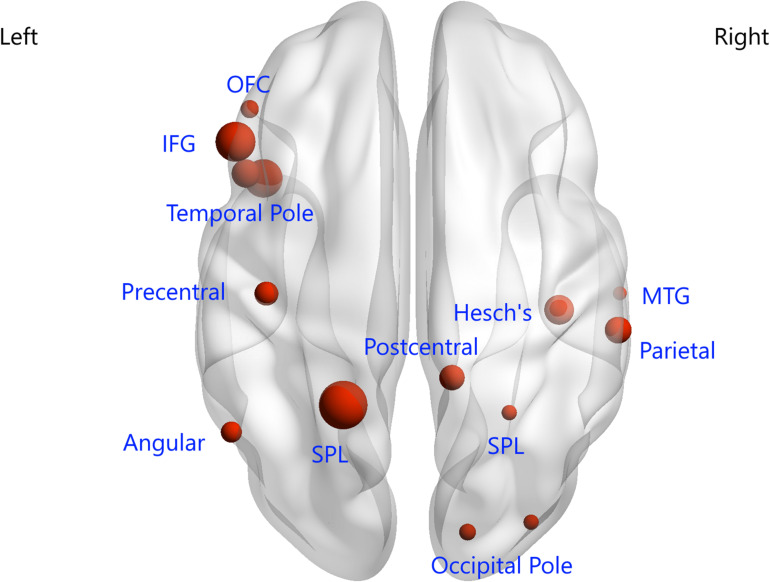
Regions of interest that contributed mostly to the accurate classification. L, left; R, right; OFC, orbital frontal cortex; IFG, inferior frontal gyrus; SPL, superior parietal lobule; MTG, middle temporal gyrus.

In this study, 397 consensus features were identified, as illustrated in Figure 2B. Eighteen regions were identified as having weights that were at least one standard deviation greater than the average of the weights of all regions. As shown in Figure 3, the ROIs making the greatest contribution to the model were located within the default mode network (DMN) (angular gyrus, middle temporal gyrus, orbital frontal gyrus, temporal pole, and inferior frontal gyrus), frontal-parietal network (superior parietal gyrus and parietal operculum cortex), auditory network (Heschl’s gyrus), and sensorimotor network (precentral gyrus and postcentral gyrus).

### Cognitive Deficits in Relatives and Correlation Analysis

**FIGURE 4:**
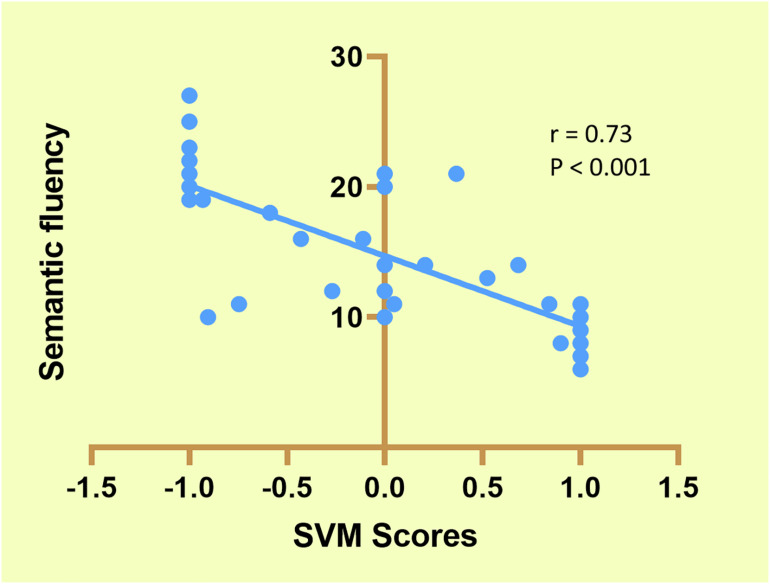
Correlation between support vector machine (SVM) scores and semantic fluency in first-degree relatives (FDRs).

As shown in Figure 4, six FDRs were given a classification score of 1 (SCZ specific) in all 76 LOOCV iterations, seven FDRs were classified as −1 (HCs specific) in all 76 LOOCV iterations, and the remaining FDRs were classified either as 1 or −1 in different iterations of the LOOCV. A significant negative correlation was found between the average classification scores of the FDRs and the semantic fluency scores.

### Classifier Performance Using Features Within the Identified Networks

**FIGURE 5:**
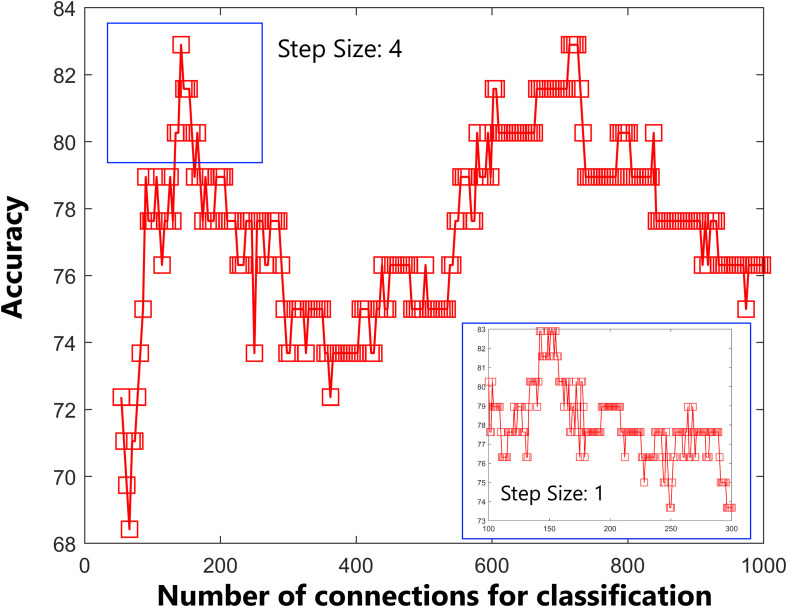
Predictive accuracy as a function of the number of connections used in the classification process. The connections were ranked according to F-scores in descending order. Classification performance was further tested by including features within the default mode network, frontal-parietal network, auditory network, and sensorimotor network only.

**FIGURE 6:**
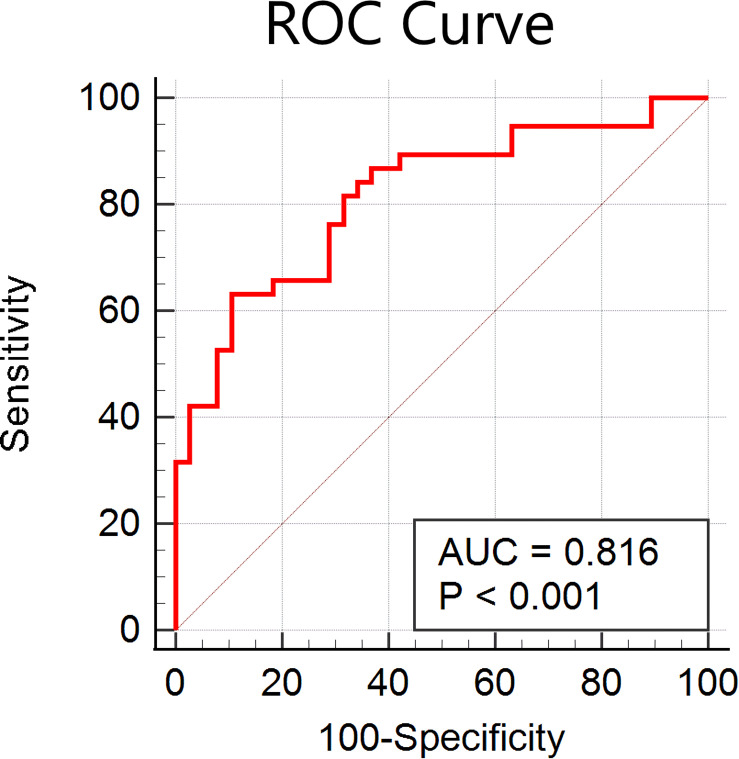
Receiver operating characteristic (ROC) curve of the classifier using features within the default mode network, frontal-parietal network, auditory network, and sensorimotor network only.

In the current study, we obtained many more features than examples (644 features were selected according to 76 participants); therefore, we reanalyzed the classifier performance only using features within the identified networks (DMN, FP, auditory, and sensorimotor). According to the networks brought up by Yeo et al. (2015), we masked the DMN, FP, auditory, and sensorimotor networks, and 142 features were obtained. As shown in Figure 5, the accuracy of the linear SVM classifier reached up to 82.89% (89.47% for specificity, 63.16% for sensitivity, and *p* < 0.001 by permutation test). The discriminative score for each tested individual was acquired from the SVM classifier, and an ROC curve was created (Figure 6), which showed an AUC of 0.82, also indicating good classification power.

## Discussion

With the use of a multivariate pattern classification method, our study demonstrated that whole-brain resting-state FC can be used to distinguish SCZ patients from HCs with excellent accuracy, with the functional connections showing the best discriminatory power being mainly located within or across the default mode, frontal-parietal, auditory, and sensorimotor networks. Furthermore, the trained machine learning model could also help to identify whether unaffected FDRs showed similar FC patterns to SCZ patients or HCs. An additional finding was that the classification scores of the unaffected FDRs correlated significantly with their word semantic fluency test scores. These findings suggest that FC, combined with a machine learning algorithm, can help to predict whether unaffected FDRs show a SCZ-specific FC pattern or a healthy control-specific FC pattern.

Cutting-edge machine learning methods have been applied in structural and functional neuroimaging studies and have revealed that multivariate patterns of brain change are sensitive enough to classify individual SCZ patients (Liu et al., 2015; Yuan et al., 2018b). Combining cortical thickness, gyrification of gray matter, and fractional anisotropy and mean diffusivity of white matter, Liang et al. (2019) used a gradient boosting decision tree to identify SCZ patients, reaching an average accuracy of 76.54%. Using global and nodal network properties derived from a graph theory analysis, Jo et al. (2020) revealed that functional network properties had a high discriminatory ability for classifying SCZ patients and HCs. Using betweenness centrality from graph theoretical approaches and a SVM algorithm, Cheng et al. (2015) found a classification accuracy of around 80% for differentiating SCZ patients from non-psychiatric HCs. Recent progress in neuroimaging research has suggested that SCZ is a dysconnectivity syndrome, and our results provide evidence that resting-state FC can be successfully used to differentiate SCZ patients from HCs.

We employed the F-score for the feature ranking in the feature selection approach, and a SVM algorithm with an LOOCV strategy showed a classification accuracy of 88.15%. We used the Craddock atlas for brain parcelation, because the brain regions are clustered in a more homogenous manner than in the AAL atlas (Craddock et al., 2012), and certain ROIs from this parcelation showed significant performance in classifying SCZ patients from HCs. Previous studies showed that an isolated brain region or connectivity dysfunction cannot be responsible for SCZ (Lefebvre et al., 2016) and indicated that impairment of interactions between several intrinsic FC networks underlies the specific psychopathological mechanism of SCZ. Our ROIs showing significant classification performance were located within previously well-studied brain networks such as the DMN, frontal-parietal network, auditory network, and sensorimotor network. Decreased communication within the DMN supports the idea of impaired self-related processes relevant to the emotional processing and recollection of prior experience (Dong et al., 2018), whereas deficits in the frontal-parietal network have been associated with poor information manipulation and poor problem-solving in goal-directed behavior, and it is proposed that abnormal interactions between the DMN and frontal-parietal network are associated with errors in the self-monitoring of SCZ (Anhoj et al., 2018; Brandl et al., 2019). Auditory network dysfunction is always found in SCZ patients, with morphological and functional abnormalities of the superior temporal gyrus, a key component of the auditory network, being frequently reported, and altered dominance in the direction of causal influence from the DMN to the auditory network also being found (Li et al., 2019). Additionally, SCZ patients have also shown impaired connectivity within sensorimotor networks, such as compromised connections between M1 and the supplementary motor area and medial motor areas (Mcnabb et al., 2018). In combination with our findings, such aberrant connectivity within and between large-scale networks not only may reflect the possible pathophysiology of SCZ but also can provide essential information, allowing us to differentiate HCs and FDRs before the development of SCZ symptoms.

First-degree relatives of patients with SCZ have an almost 10-fold increased risk of developing SCZ (Luykx et al., 2017); however, early interventions to delay or prevent the onset of psychotic disorders among these high-risk individuals have shown limited success. One of the most important hurdles is the identification of a syndrome or set of traits that reflects a predisposition to SCZ and that might provide potential targets for intervention. A neuroimaging analysis of the FDRs of patients with SCZ has mostly focused on group comparisons, with several functional brain alterations before the onset of SCZ having been reported in previous studies, although robust imaging biomarkers for the diagnosis and prediction of a later transition to psychotic disorders are still lacking. The classification scores presented in our study provided a sensitive measure for classifying FDRs as having a SCZ-specific FC pattern or HC-specific pattern, and early preventions could be provided for relatives showing a SCZ-specific pattern, especially those given labels of 1 in the LOOCV predictions. More importantly, we found that the classification scores showed a significant negative correlation with the semantic fluency scores. For those FDRs with a clear SCZ-specific pattern, psychological and psychosocial interventions (such as CBT), pharmacological interventions (such as risperidone), and nutritional supplements (such as omega-3 fatty acids) would have a beneficial effect on transition rates (Stafford et al., 2013), while for those with classification scores <+1, those interventions should be implemented with caution, as a clinically significant side effect (for example, possible increased stigma) might be induced (Stafford et al., 2013). As stated by other studies (Mourao-Miranda et al., 2012), linear SVM can effectively handle high-dimensional data and is less prone to overfitting of the data. Therefore, in this study, we exclusively used a linear kernel SVM to reduce the risk of overfitting the data and to allow direct extraction of the weight vector. The linear SVM has only one parameter (C) that controls the trade-off between having zero training errors and allowing misclassifications. This was fixed at *C* = 1 for all cases (default value). It has been shown previously that the SVM performance for whole-brain classification does not change for a large range of C values and only degrades with very small C values (Laconte et al., 2005).

Cognitive impairments are a key component of SCZ and have been included as a diagnostic criterion for SCZ in the DSM-V classification (Bortolato et al., 2015). Previous studies have indicated that non-psychotic FDRs also exhibit similar but less severe cognitive defects (Molina et al., 2016). Study of the neurocognitive functions of non-psychotic FDRs is a widely used strategy for understanding the etiology of SCZ and is free of the confounds associated with psychosis. Furthermore, it is also well accepted that cognitive impairments precede the onset of illness and represent vulnerability markers for the onset of the disorder (Viviano et al., 2018). Therefore, the negative correlations found between classification scores and cognitive performance indicate that the classifiers built on these FC measures could serve as sensitive biomarkers for the early detection of FDRs at high risk of developing SCZ.

The present study has several limitations. First, the sample size of the current study is relatively small, and the age distribution is rather young because we recruited first-episode SCZ, and a large multicenter imaging dataset containing chronic SCZ patients is necessary to confirm our findings. Second, we used cross-sectional data of the FDRs, and a longitudinal investigation is needed to verify our findings. So far, we have followed 10 unaffected FDRs for 4 years, and they remain normal. Due to low incidence rate of SCZ in unaffected FDRs, longer follow-ups are needed to investigate if the SCZ-specific FDRs had higher risk of development of the disease than the HCs-specific FDRs. Third, cognitive impairment was characterized by a small number of measures, and comprehensive measures should be collected, which may help bring neuroimaging classification scores from the bench to the bedside. Finally, we did not collect other cognitive status score such as Mini-Mental State Exam (MMSE), which would provide important information on global cognition for FDRs and HCs.

## Conclusion

Our findings indicate that brain-wide multivariate neuroimaging patterns have clear advantages for accurately classifying individuals as SCZ patients or HCs. FC within and among the default mode, frontal-parietal, auditory, and sensorimotor networks contributed most to the accurate classification. Finally, classification scores obtained by our analysis could serve as an effective and sensitive biomarker for the early detection of FDRs at high risk of developing SCZ.

## Data Availability Statement

The raw data supporting the conclusions of this article will be made available by the authors, without undue reservation.

## Ethics Statement

The studies involving human participants were reviewed and approved by the First Affiliated Hospital (Xijing Hospital) of Air Force Medical University. The patients/participants provided their written informed consent to participate in this study.

## Author Contributions

WL and XZ performed the data analysis and wrote the manuscript. YQ and YC contributed to the conception of the study. HY and MZ contributed the collection of MRI data. YZ and HW contributed to the manuscript revision. All authors read and approved the submitted version.

## Conflict of Interest

The authors declare that the research was conducted in the absence of any commercial or financial relationships that could be construed as a potential conflict of interest.
